# Plasma Adsorption with the MTx.100 Column in Critically Ill COVID-19 Patients: A Prospective Study and Propensity Score Analysis

**DOI:** 10.1177/08850666241280031

**Published:** 2024-09-12

**Authors:** Christopher Choi, Nicole De Simone, Christopher B. Webb, Peiman Lahsaei, Sean G. Yates, Jay S. Raval, Michelle S. Harkins, Donald J. Hillebrand, Antonio Belli, Nicolas A. Schlapobersky, Tina S. Ipe, Grace C. Banez-Sese, Vikramjit S. Khangoora, Steven D. Nathan, Trudy M. Demko, David C. Young, Sigalit Caron, Ravi Sarode

**Affiliations:** 1 Department of Anesthesiology and Pain Management, University of Texas Southwestern Medical Center, Dallas, TX, USA; 2 Department of Pathology, University of Texas Southwestern Medical Center, Dallas, TX, USA; 3 Carter Blood Care, Dallas, TX, USA; 4 Department of Pathology, Section of Transfusion Medicine and Therapeutic Pathology, University of New Mexico, Albuquerque, NM, USA; 5 Department of Internal Medicine, University of New Mexico, Albuquerque, NM, USA; 6 21638 Department of Gastroenterology and Hepatology, University of Kansas Medical Center, Kansas City, KS, USA; 7 Department of Neurosurgery, Queen Elizabeth Hospital, Birmingham, UK; 8 Department of Medicine and Critical Care, Christiaan Barnard Memorial Hospital, Cape Town, South Africa; 9 Department of Pathology and Laboratory Medicine, University of Arkansas for Medical Sciences, Little Rock, AR, USA; 10 Inova Therapeutic Apheresis Services and Blood Donor Services, Sterling, VA, USA; 11 23146 Inova Fairfax Hospital, Falls Church, VA, USA; 12 Department of Pulmonary and Critical Care, Reading Hospital, West Reading, PA, USA; 13 Marker Therapeutics AG, Zug, Switzerland

**Keywords:** respiratory failure, critical care, intensive care unit, lung infection, infections

## Abstract

**Background:**

Early in the COVID-19 pandemic, patients with severe disease admitted to the intensive care unit (ICU) had a high incidence of mortality. We aimed to investigate whether plasma adsorption with the MTx.100 Column could improve survival.

**Methods:**

We performed a prospective, single-arm, multicenter, Emergency Use Authorization (EUA) trial in patients admitted to the ICU with severe COVID-19 who were worsening despite standard therapy. The primary outcome was all-cause mortality on day 28. Outcomes were analyzed using both a pre-specified performance goal (PG), and a propensity score-matched (PSM) analysis from the highest enrolling center, in which patients treated with the standard of care (SOC) plus the MTx.100 Column (n = 70) were compared to a contemporaneous cohort treated at the same center with SOC only (n = 244).

**Findings:**

Between May 21, 2020, and November 2, 2021, 107 patients with severe COVID-19 (mean age 58.1) at 7 US centers were enrolled and had at least one plasma adsorption treatment initiated. All-cause mortality on day 28 was 37.4% (40/107), an improvement over the prespecified PG (88.1%, p < 0.0001). There were no serious adverse events attributable to the MTx.100 Column or plasmapheresis. Improvements in most metabolic and inflammatory markers were also noted. The PSM analysis showed that survival odds were three times higher for MTx.100 Column-treated patients (95% CI: 1.56-5.88) than for those treated with SOC only.

**Interpretation:**

The MTx.100 Column treatment in severe COVID-19 resulted in a lower mortality than SOC by both pre-specified PG and PSM analysis.

**Trial Registration:**

clinicaltrials.gov (NCT04358003).

## Introduction

The COVID-19 pandemic has resulted in over 6.9 million deaths globally and has placed a considerable strain on critical care resources worldwide.^1^ Despite the development of new therapeutics and a vast vaccination program, COVID-19 continues to be associated with a range of persistent multi-system complications in the form of ‘long-COVID’, also known as post-acute sequelae of SARS-CoV-2 infection (PASC).^2^ Particularly during the first year and a half of the pandemic, vulnerable populations developed severe respiratory sequelae ranging from viral pneumonia to acute respiratory distress syndrome (ARDS), which often necessitated admission to the intensive care unit (ICU).^3^ Other than supportive care, limited therapeutic options were available in the ICU.

In past clinical experience with severe respiratory infections, the use of therapeutic plasmapheresis to reduce the inflammatory burden, remove pathogenic components, and provide metabolic support showed some efficacy.^4,5^ Plasmapheresis involves separating whole blood into its major components, returning cellular blood components to the patient, and removing the pathogen from plasma by either replacing the patient's plasma with donor plasma or crystalloid solution (therapeutic plasma exchange [TPE]), or by plasma adsorption.

TPE, the most common form of plasmapheresis, showed early promise in treating severe COVID-19. However, TPE also reduced SARS-CoV-2-specific IgG and IgM antibody levels, which may not be desirable in an ongoing infection.^6,7^

Alternatively, in plasma adsorption therapy, plasma separated during plasmapheresis is passed through a column that adsorbs metabolic waste products and inflammatory compounds, and treated plasma is returned to the patient. Plasma adsorption eliminates the need for replacing lost blood volume with fluid during treatment, preserves the patient's immunoglobulins and electrolytes, and reduces the risk of infection and allergic reactions to donor plasma or other replacement solutions. Bench testing and several case reports have shown that the MTx.100 Plasma Adsorption Column (MTx.100 Column) removes a broad spectrum of inflammatory compounds, toxins, and metabolic wastes without removing immunoglobulins and has been associated with good clinical outcomes in severely compromised patients admitted to ICU. On April 9, 2020, the U.S. Food and Drug Administration (FDA) issued an Emergency Use Authorization (EUA 200148) for the use of the MTx.100 Column (formerly known as the Depuro D2000 Adsorption Cartridge) with the Terumo Spectra Optia Apheresis System with Secondary Plasma Device (SPD) software to treat critically ill COVID-19 patients.^8^

Results were analyzed by two methods. The prespecified performance goal (PG), 88.1%, was based on the limited information on all-cause mortality for patients admitted to the ICU with COVID-19 reported at the time of trial initiation,^9^ and was agreed on with the FDA. In the second propensity score-matched (PSM) method, we compared patients treated with the MTx.100 Column at the highest enrolling center with those treated contemporaneously at the same center.

## Methods

### Trial Design and Oversight

The EUA study of severe COVID-19 patients admitted to the ICU with a confirmed SARS-CoV-2 infection was a prospective, open-label, multicenter, single-arm study collaboratively designed with the FDA and registered on clinicaltrials.gov (NCT04358003). The study was approved by a central institutional review board (IRB), WCG (20201007), with additional local IRB approvals as required. An independent data safety monitoring board (DSMB) oversaw patient safety throughout the study. The study evaluated the safety and efficacy of treatment with the MTx.100 Column, using a prespecified performance goal based on mortality rates at the time of initiation.

Because a randomized clinical trial during the pandemic was not possible for ethical and logistical reasons, we also performed a follow-on PSM analysis. In the PSM analysis, patients treated with the MTx.100 Column under the EUA protocol were compared to a contemporary cohort of patients who were admitted to the ICU at the highest enrolling center with severe COVID-19 and treated with SOC. This analysis had a separate protocol, statistical analysis plan, and IRB approval (UTSW IRB, Protocol 2021-1048). The mortality in the two treatment arms was compared using a PSM analysis.

In the EUA study, consecutive patients admitted to the ICU with confirmed SARS-CoV-2 infection and informed consent provided by the patient or legal representative and whose disease was worsening while in the ICU were screened. Study entry criteria are in Appendix A of the Supplemental materials. The treatment protocol included once daily MTx.100 Column therapy for up to four hours (1 treatment cycle, ∼1.5 times patient plasma volume) for four consecutive days, with the option to treat longer if deemed necessary by the investigator. The Terumo Spectra Optia Apheresis System with SPD software (manufactured by Terumo BCT) was used to separate patient plasma from whole blood and plasma was passed through the MTx.100 Column; cellular blood components and adsorbed plasma were then reinfused to the patient. Technical information on the apheresis procedure is given in Appendix B of the Supplemental materials. Before initiating the daily treatment cycle, pre-treatment chemistry, hematology, coagulation parameters, and disease severity scores (SOFA, APACHE II) were collected. Post-therapy lab parameters were collected immediately following each therapy. Serious adverse events (SAEs) were collected during the treatment phase (starting at the first treatment through the last treatment). Patients were followed for 28 days.

For the PSM analysis, propensity scores were calculated using the following variables: age quartiles, race, BMI category, ventilator status, history of cardiac disease, pulmonary disease, diabetes, hypertension, solid organ transplant, and date of hospital admission (quarter and year).

### Statistical Analysis

The statistical analysis was performed using SAS. The PG (88.1%), which was agreed on with the FDA, was based on all-cause mortality for ICU patients reported early in the pandemic.^9^ The primary propensity score analysis was a Chi-square test of mortality rates using stabilized inverse-propensity score weights. Mortality rates changed during the pandemic, therefore, two sensitivity analyses were also performed to use a contemporaneous comparison group: (1) matched pairs/triplets (1 MTx.100 Column-treated patient and 1 or 2 contemporary SOC-treated patients) and (2) propensity-score quartiles. Matched pairs/triplets were created using the greedy algorithm to yield pairs or triplets with similar propensity scores. The gmatch macro in SAS matched patients in a 1:2 ratio (MTx.100 Column: SOC) based on similar propensity scores. A window of ±0.10 was used for the matching; if a SOC patient with a propensity score within 0.10 of the MTx.100 patient could not be found, that MTx.100 patient was not included in the matched pairs/triples analyses. For some MTx.100 patients, only one SOC patient was found within the propensity score window; these comprise the matched pairs. The matched pairs/triples were analyzed using a Cochran-Mantel-Haenszel test, stratified by pair/triple number. In the propensity-score quartile analysis, all patients (MTx.100 and SOC patients) were grouped based on their propensity scores. Mortality was then compared between the two treatment groups in a Cochran-Mantel-Haenszel test, stratified by propensity-score quartile.

## Results

### Baseline Patient Characteristics

A total of 107 patients were enrolled at 7 centers and initiated at least one MTx.100 Column treatment between May 21, 2020, and November 2, 2021. Demographics and baseline characteristics are summarized in Table 1. Seventy percent (75/107) of patients were on an invasive form of ventilation at screening and 11% of patients on invasive ventilation were also on ECMO (8/75).

**Table 1.** Patient Demographics and Baseline Characteristics of the Treated Population (N = 107).

Demographics and baseline characteristics	Number*
**Age (years)**	58.1 ± 13.2 (107) 60 (50, 67) [17, 82]
< 50 years	25 (23.4%)
50 to <65 years	48 (44.9%)
65 to <85 years	34 (31.8%)
**Sex**	
Female	38 (35.5%)
Male	69 (64.5%)
**Race**	
African American	19 (17.8%)
Asian	4 (3.7%)
Native American	4 (3.7%)
White	72 (67.3%)
Other (unknown)	8 (7.5%)
**Ethnicity**	
Hispanic or Latino	40 (37.4%)
Not Hispanic or Latino	66 (61.7%)
No Response/Missing	1 (0.9%)
**Confirmed COVID Diagnosis**	107 (100.0%)
**Ventilatory Support at Screening**	
Invasive	75 (70.1%)
Non-invasive	32 (29.9%)

* *Continuous measures - Mean ± SD (total number), median (Q1, Q3), [min, max]; Categorical measures - n (%).*

The mean number of comorbidities reported for each patient was 6.2 (SD 4.1, range 1-22 comorbidities). Nearly half of the study population (49.5%) had ≥ 6 comorbidities (Table 2). The most common pre-defined comorbidity was hypertension (65.4%), followed by diabetes (43.9%), cardiovascular disease (24.3%), and chronic kidney disease (21.5%; Table 3).

**Table 2.** Number of Comorbidities in the Treated Population (N = 107).

Number of comorbidities	Number of patients*
**Overall population**	6.2 ± 4.1 (107) 5 (3, 8) [1, 22]
1	8 (7.5%)
2	9 (8.4%)
3	14 (13.1%)
4	14 (13.1%)
5	9 (8.4%)
≥ 6	53 (49.5%)

* *Continuous measures - Mean ± SD (total number), median (Q1, Q3), [min, max]; Categorical measures - n (%)*

**Table 3.** Pre-Defined Comorbidities in the Treated Population (N = 107).

Pre-defined comorbidity	Number (%)
Hypertension	70 (65.4%)
Diabetes	47 (43.9%)
Cardiovascular disease	26 (24.3%)
Chronic kidney disease	23 (21.5%)
Chronic obstructive pulmonary disease	9 (8.4%)
Cancer	6 (5.6%)
Cerebrovascular disease	4 (3.7%)
Chronic liver disease	4 (3.7%)
Smoking	3 (2.8%)

The sites were able to report “other” comorbidities as well. The most common “other” comorbidity was obesity (57.0%), followed by hyperlipidemia (30.8%), gastroesophageal reflux disease (22.4%), and sleep apnea (21.5%). The comprehensive list of all comorbidities is in Appendix C of the Supplemental materials.

### Treatment with the MTx.100 Column

Plasma adsorption with the MTx.100 Column was well tolerated. A total of 424 treatments were administered. The mean number of treatments per patient was 4.0 (SD 1.1), and 70% (n = 75) received 4 treatments. The mean total blood volume or plasma volume treated per cycle was 5216 mL (SD 950 mL, range of 3135 mL to 7635 mL). A total of 15 device deficiencies were reported; 3 device deficiencies involved the Spectra Optia Apheresis System with SPD. In 9 of these 12 instances, the column was replaced, and treatment was completed. None of the device deficiencies resulted in an SAE.

### Efficacy

All-cause mortality at day 28 for the intention to treat population (all subjects exposed to the column) was 37.4% (40/107) and was significantly lower than the prespecified PG of 88.1% (p < 0.0001) based on an exact binomial test. This was despite the fact that 9.4% (10/107) of patients were in such critical condition at the initiation of the first treatment that they expired before the completion of the first treatment. The causes of death were categorized as COVID-19 (n = 19), cardiopulmonary arrest (n = 7), respiratory failure (n = 6), multi-organ failure (n = 4), cardiogenic shock (n = 3) and pulmonary embolism (n = 1). Similar mortality rates (∼37%) were reported by race (white vs non-white). Patients on invasive ventilation had a higher mortality than those not on invasive ventilation: 42.7% and 25.0%, respectively.

SOFA and APACHE II scores from baseline to last treatment were highly variable and did not follow a meaningful trend (Appendix D, Supplemental materials). Laboratory parameters such as mean levels of bilirubin, creatinine, CRP, LDH, BUN, and fibrinogen decreased from baseline (prior to the first treatment) to after the last treatment, a known effect of treatment with the MTx.100 Column (Appendix D, Supplemental materials). Cytokine data were available for between 74 and 78 patients for IL-1β, IL-4, IL-6, IL-8, IL-10 and TNFα. The average baseline levels for all cytokines (except for IL-1β) showed extreme elevations, above the upper limit of the reference range. These levels for most of these cytokines decreased, albeit variably, after adsorption (Appendix D, Supplemental materials).

### Safety

There were no unanticipated adverse device effects (UADEs). A total of 38 SAEs were reported for 27 patients (25.3%) and consisted of 10 SAEs (26.3%) that were reported during the plasma adsorption phase, 26 SAEs (68.4%) that resolved, and 2 SAEs (5.3%, involving oxygen desaturation and deep vein thrombosis) that were ongoing at study exit. Four patients (2 patients each at 2 sites) experienced 9 incidents of fluid volume overload which resolved without sequelae. There were no incidents of allergic reactions, acute immune responses or immunosuppression, or other ICU-acquired infections. Appendix E in the Supplemental materials lists all SAEs.

### Propensity Score-Matched Analysis

Throughout the first year of the pandemic, mortality rates steadily declined for multiple reasons. SOC improved (partly owing to the use of additional therapeutics), and patients admitted to the ICU tended to be younger and have a lower SOFA score.^10–12^ To better understand the therapeutic impact of the MTx.100 Column, we undertook a PSM analysis to compare patients treated with the MTx.100 Column with contemporaneously treated patients with similar characteristics, which may be more relevant than either the historical PG or statistics from multiple ICUs (Table 4).

**Table 4.** Propensity score-Matched Analysis.

Analysis	Treatment group		Statistical calculation	
	MTx.100	SOC	Odds ratio	P-value
**Primary endpoint analysis**				
*N*	70	244		
Survival	0.80	0.57	3.02	0.0008
95% CI	(0.68, 0.88)	(0.50, 0.63)	(1.56, 5.85)	
**Sensitivity analyses**				
**Matched pairs/triples**				
*N*	66	104		
Survival	0.73	0.46	2.97	<0.0001
95% CI	(0.61, 0.82)	(0.37, 0.56)	(1.53, 5.76)	
**Propensity score quartiles**				
*N*	70	244		
Survival	0.71	0.59	2.57	0.002
95% CI	(0.60, 0.81)	(0.53, 0.65)	(1.35, 4.91)	

Although 7 centers enrolled patients, the majority (70) were enrolled at University of Texas Southwestern Medical Center (UTSW). These 70 patients treated at UTSW under the EUA protocol were compared to 244 SOC patients who were selected to have similar baseline characteristics and who were treated at UTSW over the same time period (Appendix F, Supplemental materials). Enrolment in the EUA trial required patients to be rapidly progressing despite SOC; the PSM-matched patients were admitted to the ICU during the same time period but were not enrolled in the trial. All patients had 28-day all-cause mortality data and hospital length-of-stay data. Demographics (age, sex, race, ethnicity, BMI) in the two treatment groups were similar (t-test or Fisher's exact p-values > 0.05), but more patients in the MTx.100 Column group were on ventilators than in the SOC group (87.1% vs 55.7%) and more MTx.100 Column-treated patients were enrolled in the last quarter of 2020 and 2021 (80.0%) than SOC (43.8%), while more SOC patients were enrolled in the first 3 quarters of 2020. These two differences may be related: patients were only admitted to the ICU at UTSW in the later months (Q4 2020 on) if intubation or nitrous oxide were likely to be needed. Comorbidity rates for cardiac disease, pulmonary disease, diabetes, hypertension, and history of solid organ transplant were similar for the two groups (Fisher's exact p-values > 0.05). All demographics, comorbidities, ventilator status and dates of admission were similar across the MTx.100 Column-treated and SOC groups when compared in the propensity-matched pairs/triples (p-values > 0.05), indicating that the propensity score matching adjusted for the imbalance in ventilator status and date of admission.

In the stabilized inverse-propensity score weighted analysis, the mortality was significantly lower for the MTx.100 Column-treated patients (20%) than for SOC patients (43%) (p-value = 0.0008). The odds of survival were 3.0 times higher for MTx.100 Column-treated patients (95% CI: 1.56-5.85) than for SOC patients. These results were supported in the sensitivity analyses of propensity-score matched pairs/triples (odds ratio = 3.0) and propensity-score stratified analysis (odds ratio = 2.6). For patients that survived at least 30 days, hospital stays were significantly longer for MTx.100 Column-treated patients, by approximately 12 days (p-value = 0.01), as these patients remained hospitalized during convalescence. Likely because enrolment in the EUA trial required patients to be progressing despite therapy, and did not preclude additional therapies, MTx.100 Column-treated patients were more likely to be treated with steroids (p-value = 0.03), JAK inhibitors (p-value < 0.0001), remdesivir (p-value < 0.0001), and convalescent plasma (p-value < 0.0001).

## Discussion

The overall 28-day mortality for patients treated with the MTx.100 Column was 37.4%, which was significantly lower than the prespecified PG (88.1%, p < 0.0001). From our PSM analysis, mortality was significantly lower in patients treated with the MTx.100 Column than in those treated with SOC (p = 0.0008).

The alpha and delta SARS-CoV-2 variants (identified in May of 2020 and December of 2020, respectively), which were associated with serious complications, predominated during the enrollment period for this study.^13^ Patients admitted to the ICU and placed on mechanical ventilation during these waves experienced high mortality rates.^9,14^

We initially hypothesized that treatment with the MTx.100 Column would be effective largely through its demonstrated ability to remove cytokines from the circulation. Although substantial amounts of cytokines were removed, patients alive at day 28 still had average IL-6 levels 21 times the upper limit of normal, suggesting that the removal of cytokines likely does not solely account for the reduced mortality. Importantly, all treated patients had undetectable or low, normal IL-1β levels and extremely high levels of IL-6 immediately prior to their first treatment. Other studies in COVID-19 patients admitted to hospital documented an early and transient elevation in IL-1β levels,^15^ and this cytokine is known to peak early during many infections. Therefore, the low IL-1β levels suggest that the beginning of the inflammatory cytokine storm had long since passed, and that organ damage and a dysregulated runaway immune response had taken hold prior to the first treatment with the MTx.100 Column.

We therefore postulate that the reduced mortality in MTx.100 Column-treated patients may be multifaceted: a combination of removing a broad spectrum of inflammatory compounds, which interrupts the hyperinflammation cycle, and removing a substantial proportion of metabolic waste products (such as creatinine and BUN), which reduces the strain on the liver and kidneys. For severely ill patients, removing these waste products may be sufficient to interrupt the deterioration of their overall health and organ function.

Between the initiation of our study and the submission of this manuscript, multiple studies of TPE have been conducted in patients with COVID-19. A recent meta-analysis examining patients from 13 trials, 485 of whom received TPE, found evidence that the use of TPE, compared with SOC, improves metabolic and inflammatory markers and is associated with a ∼20% reduction in mortality.^16^ Although most of this evidence was of low or moderate quality and direct comparisons cannot be made between TPE and plasma adsorption or between these trials and ours, the collective results suggest that removing inflammatory compounds and waste products from blood is beneficial to patients with COVID-19. There have been no new reports of plasma adsorption therapy in patients with COVID-19 since the initiation of our study. The possible advantages of column therapy over TPE are that there is selective adsorption of cytokines and toxic waste products rather than removal of patient plasma (which contains antibodies that are important in critically ill patients), and column therapy also avoids giving donor plasma, which could be detrimental to already damaged lungs.

A limitation of the study was that it was a single-arm study with no direct comparator arm, as dictated by the pandemic constraints in the ICU setting at that time. Enrolment in the trial required patients to be worsening despite SOC; treatment with the MTx.100 Column was thus a last resort for most patients. Patients treated with the MTx.100 Column arm were therefore likely to be more ill than average.

Owing to these limitations, a retrospective PSM analysis was conducted. Although this analysis provides a clinical context for interpreting the results, matching the SOC controls was based on EUA-identified criteria, and cytokine levels and other metabolic data were not collected for both groups. The PSM analysis only adjusted for the variables that were included (ie, age, sex, etc). There was no ability to account for differences in the use of medications such as JAK2 inhibitors and steroids, but one can reasonably assume that at least some of the patients were already on long-term steroid therapy or required steroids for other indications.

## Conclusion

Plasma adsorption with the MTx.100 Column in critically ill COVID-19 patients in an ICU setting resulted in significant survival benefits when compared to PSM controls. Unlike some drugs or interventional therapies used during the first year of the pandemic, the use of the MTx.100 Column was not associated with adverse immune responses, immunosuppression, or increases in other ICU-acquired infections. Its safety, even in a critically ill patient population, suggests that a study of earlier intervention with this therapeutic approach may be warranted, and could be considered if novel pathogens emerge in the future. In addition, further trials should explore therapy with the MTx.100 Column in hyperinflammatory conditions.

## Supplemental Material

sj-docx-1-jic-10.1177_08850666241280031 - Supplemental material for Plasma Adsorption with the MTx.100 Column in Critically Ill COVID-19 Patients: A Prospective Study and Propensity Score Analysis Supplemental material, sj-docx-1-jic-10.1177_08850666241280031 for Plasma Adsorption with the MTx.100 Column in Critically Ill COVID-19 Patients: A Prospective Study and Propensity Score Analysis by Christopher Choi, Nicole De Simone, Christopher B. Webb, Peiman Lahsaei, Sean G. Yates, Jay S. Raval, Michelle S. Harkins, Donald J. Hillebrand, Antonio Belli, Nicolas A. Schlapobersky, Tina S. Ipe, Grace C. Banez-Sese, Vikramjit S. Khangoora, Steven D. Nathan, Trudy M. Demko, David C. Young, Sigalit Caron and Ravi Sarode in Journal of Intensive Care Medicine

## References

[1] Ndayishimiye C Sowada C Dyjach P , et al. Associations between the COVID-19 pandemic and hospital infrastructure adaptation and planning—a scoping review. Int J Environ Res Public Health. 2022;19(13):8195. 35805855 10.3390/ijerph19138195 PMC9266736

[2] Davis HE McCorkell L Vogel JM Topol EJ . Long COVID: Major findings, mechanisms and recommendations. Nat Rev Microbiol. 2023;21(3):133-146. 36639608 10.1038/s41579-022-00846-2 PMC9839201

[3] Ribeiro Carvalho CR Lamas CA Chate RC , et al. Long-term respiratory follow-up of ICU hospitalized COVID-19 patients: Prospective cohort study. PloS one. 2023;18(1):e0280567. 10.1371/journal.pone.0280567 PMC9858876 36662879

[4] Patel P Nandwani V Vanchiere J Conrad SA Scott LK . Use of therapeutic plasma exchange as a rescue therapy in 2009 pH1N1 influenza A—an associated respiratory failure and hemodynamic shock. Pediatric Critical Care Medicine: A Journal of the Society of Critical Care Medicine and the World Federation of Pediatric Intensive and Critical Care Societies. 2011;12(2):e87. 10.1097/PCC.0b013e3181e2a569 PMC6328374 20453703

[5] Connelly-Smith L Alquist CR Aqui NA , et al. Guidelines on the use of therapeutic apheresis in clinical practice–evidence-based approach from the writing committee of the American society for apheresis: The ninth special issue. J Clin Apher. 2023;38(2):77-278. 37017433 10.1002/jca.22043

[6] Beraud M Al Hashami S Lozano M Bah A Keith P . Role of therapeutic plasma exchange in the management of COVID-19-induced cytokine storm syndrome. Transfus Apher Sci. 2022;61(4):103433. 35341691 10.1016/j.transci.2022.103433 PMC8942460

[7] Stahl K Bode C David S . First do no harm—beware the risk of therapeutic plasma exchange in severe COVID-19. Crit Care. 2020;24(1):1-2. 32552832 10.1186/s13054-020-03070-7 PMC7301767

[8] www.federalregister.gov/documents/2020/06/05/2020-12117/authorization-of-emergency-use-of-certain-medical-devices-during-covid-19-availability#citation-9-p34641

[9] Richardson S Hirsch JS Narasimhan M , et al. Presenting characteristics, comorbidities, and outcomes among 5700 patients hospitalized with COVID-19 in the New York city area. JAMA. 2020;323(20):2052-2059. 32320003 10.1001/jama.2020.6775 PMC7177629

[10] Garcia Borrega J Naendrup J-H Heindel K , et al. Clinical course and outcome of patients with SARS-CoV-2 alpha variant infection compared to patients with SARS-CoV-2 wild-type infection admitted to the ICU. Microorganisms. 2021;9(9):1944. 34576839 10.3390/microorganisms9091944 PMC8470850

[11] Greco M De Corte T Ercole A , et al. Clinical and organizational factors associated with mortality during the peak of first COVID-19 wave: The global UNITE-COVID study. Intensive Care Med. 2022;48(6):690-705. 35596752 10.1007/s00134-022-06705-1 PMC9123859

[12] Wendel-Garcia P Moser A Jeitziner M , et al. Dynamics of disease characteristics and clinical management of critically ill COVID-19 patients over the time course of the pandemic: An analysis of the prospective, international, multicentre RISC-19-ICU registry. Crit. Care. 2022;26(1):199. 35787726 10.1186/s13054-022-04065-2 PMC9254551

[13] Khatri R Siddqui G Sadhu S , et al. 2022. SARS-CoV-2 variants’-Alpha, Delta, and Omicron D614G and P681R/H mutations impact virus entry, fusion, and infectivity. 2022. 10.1007/s00430-022-00760-7 PMC9801140 36583790

[14] Lim ZJ Subramaniam A Ponnapa Reddy M , et al. Case fatality rates for patients with COVID-19 requiring invasive mechanical ventilation. A meta-analysis. Am J Respir Crit Care Med. 2021;203(1):54-66. 33119402 10.1164/rccm.202006-2405OC PMC7781141

[15] Ong EZ Chan YFZ Leong WY , et al. A dynamic immune response shapes COVID-19 progression. Cell Host Microbe. 2020;27(6):879-882.e2. 32359396 10.1016/j.chom.2020.03.021 PMC7192089

[16] Porosnicu TM Sirbu IO Oancea C , et al. The impact of therapeutic plasma exchange on inflammatory markers and acute phase reactants in patients with severe SARS-CoV-2 infection. Medicina (B Aires). 2023;59(5):867. 10.3390/medicina59050867 PMC10221344 37241099

