# Supplementary material for: Plasma Adsorption with the MTx.100 Column in Critically Ill COVID-19 Patients: A Prospective Study and Propensity Score Analysis
Source: J Intensive Care Med. 2024 Sep 12;40(3):314–9. doi: 10.1177/08850666241280031 (PMC11915769; doi:10.1177/08850666241280031)
Supplement: sj-docx-1-jic-10.1177_08850666241280031 - Supplemental material for Plasma Adsorption with the MTx.100 Column in Critically Ill COVID-19 Patients: A Prospective Study and Propensity Score Analysis [file sj-docx-1-jic-10.1177_08850666241280031.docx]

**Supplementary Materials**

**Appendix A – Entry criteria**

| **Inclusion**  Subjects had to satisfy all of the following inclusion criteria to be included in the study:   1. Age ≥ 18 years old 2. Admitted to ICU 3. Diagnosis of SARS-CoV-2 with any one of the following conditions:    1. Early acute lung injury (ALI)/early acute respiratory distress syndrome (ARDS); or    2. Severe disease, defined as:       1. dyspnea,       2. respiratory frequency ≥ 30/min,       3. blood oxygen saturation ≤ 93%,       4. partial pressure of arterial oxygen to fraction of inspired oxygen ratio < 300, and/or       5. lung infiltrates > 50% within 24 to 48 hours; or    3. Life-threatening disease, defined as:       1. respiratory failure,       2. septic shock, and/or       3. multiple organ dysfunction or failure. 4. Patient fact sheet is provided to the subject. 5. Subject or legal representative is able and willing to give informed consent. If authorized by the IRB, emergent plasma adsorption with the D2000 cartridge may be initiated prior to consent.   **Exclusion**  Subjects ***were not eligible for the study*** if any of the following criteria were present:   1. Treatment limitation or a do not attempt to resuscitate in place 2. Pregnancy 3. Significant or uncontrolled bleeding 4. In the opinion of the investigator, any other condition that precludes plasma adsorption with the D2000. |
| --- |

**Appendix B - Technical details for performing plasma adsorption with the MTx.100 secondary plasma device**

Apheresis procedures were performed using the Spectra Optia® Apheresis System (Terumo BCT, Lakewood, CO) using a standard therapeutic plasma exchange kit and tubing set with a secondary plasma device (SPD), the MTx.100 Plasma Adsorption Column (MTx.100; Marker AG, Zug, Switzerland). Vascular access was either a double lumen apheresis compatible catheter or extracorporeal device tubing. The citrate was used as an anticoagulant at a default AC ratio of 12:1, unless adjustment was needed. The MTx.100 was primed with a 200 mL of normal saline, gently rocked approximately ~20 times to distribute the adsorption media uniformly within the column, then rinsed with an additional 800 ml. The divert prime volume was set to 150 ml, replace pump balance was set to 100%, and plasma and inlet flow rates were set to a maximum of 50 mL/min. 1.0-1.5 plasma volumes were processed with each procedure. Upon initiating the apheresis procedure, the plasma pump transferred treated plasma from the MTx.100 into a transfer bag that contained up to a total of 500 mL or 5% of the patient's TBV, whichever was smaller. The replace pump then began continuously returning treated plasma to the patient. At the end of the procedure, 200 mL of normal saline could be pumped through the tubing set to rinse treated plasma from the column. The cellular blood components (red blood cells and buffy coat) were then rinsed back to the patient. Returning any residual treated plasma within the transfer bag was also an option. Due to concern for volume overload in these patients, most had neither the MTx.100 final rinse nor return of any residual treated plasma.

**General strategies implemented in the treatment of therapeutic apheresis patients in the setting of airborne isolation**

As plasma adsorption was performed in critically ill patients with COVID-19 infections, the procedures were performed using strategies implemented earlier during the pandemic to provide apheresis care while ensuring nurse, provider, and equipment safety. Continuous exposure to patients in airborne isolation increases the risk of disease transmission and instrument contamination, and thus limiting bedside contact was a priority. Due to the need for continued monitoring, fluid spiking, and other interventions, the apheresis instrument was kept outside of the patient room.^1^ The inlet line was extended using blood warmer tubing that added an additional 42 mL of extracorporeal volume to the circuit. For each apheresis procedure performed in this fashion, a two-nurse team was involved. The apheresis nurse was responsible for operating and monitoring the apheresis machine outside the room. The second ICU nurse was responsible for accessing the patient and monitoring the patient during the start and end of the procedure. After monitoring the procedure for the first 15 minutes, the ICU nurse left the room and treatment was monitored by the nurse outside the room. Once the rinseback was completed, the ICU nurse entered the room to disconnect and perform vascular access care while the apheresis nurse oversaw end-of-procedure instrument care and cleaning.

**Supplementary reference:**

1. Reyes C, Ornelas CJ, Rollins‐Raval MA, Subbaswamy AV, Phillips JP, Raval JS. A strategy to conserve personal protective equipment while performing therapeutic plasma exchange in a patient with COVID‐19. *Journal of Clinical Apheresis* 2020; **35**(4): 374.

**Appendix C - Comorbidities**

**Non-predefined comorbidities in the treated population (*N* = 107)**

| **Comorbidity** | **Number of patients (%)** |
| --- | --- |
| Obesity | 61 (57.01%) |
| Hyperlipidemia | 33 (30.84%) |
| Gastroesophageal reflux disease | 24 (22.43%) |
| Sleep apnea | 23 (21.50%) |
| Anemia | 17 (15.89%) |
| Arrhythmia | 13 (12.15%) |
| Depression | 13 (12.15%) |
| Asthma | 10 (9.35%) |
| Anxiety | 9 (8.41%) |
| Arthritis | 9 (8.41%) |
| Deep vein thrombosis | 8 (7.48%) |
| Hypothyroidism | 8 (7.48%) |
| Back pain | 7 (6.54%) |
| Acute kidney injury | 6 (5.61%) |
| Benign prostatic hypertrophy | 6 (5.61%) |
| Allergies | 5 (4.67%) |
| Gout | 5 (4.67%) |
| Heart failure | 5 (4.67%) |
| Osteopenia | 5 (4.67%) |
| Pulmonary embolism | 5 (4.67%) |
| Rhinitis | 5 (4.67%) |
| Seizures | 5 (4.67%) |
| Glaucoma | 4 (3.74%) |
| Heart transplantation | 4 (3.74%) |
| Lung disease | 4 (3.74%) |
| Osteoarthritis | 4 (3.74%) |
| Pneumonia | 4 (3.74%) |
| Acute respiratory distress syndrome | 3 (2.80%) |
| Dementia | 3 (2.80%) |
| Diverticulitis | 3 (2.80%) |
| Gastrointestinal bleeding | 3 (2.80%) |
| Headache | 3 (2.80%) |
| Hypercholesterolemia | 3 (2.80%) |
| Hyperparathyroidism | 3 (2.80%) |
| Neuropathy | 3 (2.80%) |
| Osteoporosis | 3 (2.80%) |
| Renal failure | 3 (2.80%) |
| Actinic keratosis | 2 (1.87%) |
| Ankylosing spondylitis | 2 (1.87%) |
| Attention deficit hyperactivity disorder | 2 (1.87%) |
| Bilateral lung transplant | 2 (1.87%) |
| Chest tightness | 2 (1.87%) |
| Confusion | 2 (1.87%) |
| Cough | 2 (1.87%) |
| Encephalopathy | 2 (1.87%) |
| Hypokalemia | 2 (1.87%) |
| Hypoxia | 2 (1.87%) |
| Kidney transplant | 2 (1.87%) |
| MRSA infection | 2 (1.87%) |
| Myalgia | 2 (1.87%) |
| Myasthenia gravis | 2 (1.87%) |
| Peripheral artery disease | 2 (1.87%) |
| Pneumomediastinum | 2 (1.87%) |
| Septic shock | 2 (1.87%) |
| Transaminitis | 2 (1.87%) |
| Urinary tract infection | 2 (1.87%) |
| Vitamin D deficiency | 2 (1.87%) |
| Weakness | 2 (1.87%) |
| Acidosis | 1 (0.93%) |
| Adrenal nodules | 1 (0.93%) |
| Agitation | 1 (0.93%) |
| Alcohol withdrawal syndrome | 1 (0.93%) |
| Aortic valve disease | 1 (0.93%) |
| Autonomic dysreflexia | 1 (0.93%) |
| Avascular necrosis of hips | 1 (0.93%) |
| Azotemia | 1 (0.93%) |
| Bacteremia | 1 (0.93%) |
| Bipolar affective disorder | 1 (0.93%) |
| Blind eye removal | 1 (0.93%) |
| C. difficile colitis | 1 (0.93%) |
| Cardiac arrest | 1 (0.93%) |
| Cardiogenic shock | 1 (0.93%) |
| Cardiomegaly | 1 (0.93%) |
| Cardiomyopathy | 1 (0.93%) |
| Cerebral aneurysm | 1 (0.93%) |
| Cold agglutinin disease | 1 (0.93%) |
| Colon polyps | 1 (0.93%) |
| Congenital heart defect | 1 (0.93%) |
| Constipation | 1 (0.93%) |
| Diverticulosis | 1 (0.93%) |
| Dysphagia | 1 (0.93%) |
| Elevated PSA | 1 (0.93%) |
| Fatty liver | 1 (0.93%) |
| Fibromyalgia | 1 (0.93%) |
| Gastritis | 1 (0.93%) |
| Heartburn | 1 (0.93%) |
| Hematuria | 1 (0.93%) |
| Hemoptysis | 1 (0.93%) |
| Hepatitis | 1 (0.93%) |
| Hepatosplenomegaly | 1 (0.93%) |
| Herpes | 1 (0.93%) |
| Herpes zoster | 1 (0.93%) |
| Hip pain | 1 (0.93%) |
| Hyperglycemia | 1 (0.93%) |
| Hypersomnia | 1 (0.93%) |
| Hyperthyroidism | 1 (0.93%) |
| Hypoalbuminemia | 1 (0.93%) |
| Hypocalcemia | 1 (0.93%) |
| Hypogammaglobulinemia | 1 (0.93%) |
| Hypoparathyroidism | 1 (0.93%) |
| Hypotension | 1 (0.93%) |
| Immunosuppression | 1 (0.93%) |
| Increased bilirubin | 1 (0.93%) |
| Inflammatory bowel disease | 1 (0.93%) |
| Iritis | 1 (0.93%) |
| Jaundice | 1 (0.93%) |
| Legally blind | 1 (0.93%) |
| Leukopenia | 1 (0.93%) |
| Low testosterone | 1 (0.93%) |
| Lymphopenia | 1 (0.93%) |
| Multiple sclerosis | 1 (0.93%) |
| Musculoskeletal disease | 1 (0.93%) |
| Myocarditis | 1 (0.93%) |
| Neurocognitive disorder | 1 (0.93%) |
| Neurocysticercosis | 1 (0.93%) |
| Neurogenic bladder | 1 (0.93%) |
| Overactive bladder | 1 (0.93%) |
| Peripheral vascular disease | 1 (0.93%) |
| Peritonitis | 1 (0.93%) |
| Pituitary adenoma | 1 (0.93%) |
| Pneumothorax | 1 (0.93%) |
| Polycystic kidney disease | 1 (0.93%) |
| Polycystic liver disease | 1 (0.93%) |
| Polycystic ovarian disease | 1 (0.93%) |
| Postoperative bleeding | 1 (0.93%) |
| Priapism | 1 (0.93%) |
| Pseudo pneumothorax | 1 (0.93%) |
| Pseudoaneurysm | 1 (0.93%) |
| Psoriasis | 1 (0.93%) |
| Pulmonary fibrosis | 1 (0.93%) |
| Pulmonary hypertension | 1 (0.93%) |
| Pyelonephritis | 1 (0.93%) |
| Radial artery thrombosis | 1 (0.93%) |
| Restless leg syndrome | 1 (0.93%) |
| Right ventricular dysfunction | 1 (0.93%) |
| Sarcoidosis | 1 (0.93%) |
| Schizoaffective disorder | 1 (0.93%) |
| Sepsis | 1 (0.93%) |
| Sjogren's syndrome | 1 (0.93%) |
| Small airway disease | 1 (0.93%) |
| Spinal fracture | 1 (0.93%) |
| Stem cell transplant | 1 (0.93%) |
| Stroke | 1 (0.93%) |
| Subarachnoid hemorrhage | 1 (0.93%) |
| Thalassemia | 1 (0.93%) |
| Thrombocytopenia | 1 (0.93%) |
| Thyroid disease | 1 (0.93%) |
| Tracheal stenosis | 1 (0.93%) |
| Uterine artery embolization | 1 (0.93%) |
| Vaping | 1 (0.93%) |
| Vasculopathy | 1 (0.93%) |
| Venous stasis | 1 (0.93%) |
| Vertigo | 1 (0.93%) |
| Vitamin B12 deficiency | 1 (0.93%) |

**Appendix D – SOFA & APACHE II scores, cytokine data & other laboratory parameters**

***SOFA & APACHE II scores.*** The mean SOFA score was 8.0 ± 3.8 prior to the first treatment (baseline) and 8.6 ± 3.7 after the last treatment. The change in SOFA score from baseline to the last treatment was 0.5 ± 0.5. The mean APACHE II score was 19.8 ± 6.7 prior to the first treatment (baseline) and 20.5 ± 7.4 after the last treatment. The change in mean APACHE II score from baseline to after the last treatment was 1.1 ± 6.5.

***Cytokines***. Plasma samples were processed at the site and batch shipped to the core laboratory (Charles River Laboratories located in Montreal, Canada). However, due to the variability and patient to patient inconsistency of the cytokine analysis, this data collection was terminated prior to study closeout. Pre-treatment day 1 cytokine results for patients with corresponding day 4 results are presented in Table 1. Day 4 data for these patients are presented in Table 2, and changes are presented in Table 3.

**Table 1. Day 1 cytokine data (safety population)**

| **Lab parameter (pg/mL)** | **Mean** | **St. Dev.** | **Min.** | **Max.** | **Median** | ***N*** | **Reference value**^†^ |
| --- | --- | --- | --- | --- | --- | --- | --- |
| IL-10 | 42.5 | 62.1 | 5.9 | 377.5 | 26.3 | 56 | ≤ 6.7 |
| IL-1β | 20.6 | 142.1 | 1.0 | 1074.6 | 1.0 | 57 | ≤ 2.2 |
| IL-4 | 58.9 | 113.1 | 7.3 | 779.9 | 29.0 | 57 | ≤ 2.0 |
| IL-6 | 36.3 | 88.7 | 0.7 | 636.7 | 14.4 | 55 | ≤ 3.0 |
| IL-8 | 32.6 | 30.1 | 2.5 | 140.8 | 19.7 | 55 | ≤ 2.8 |
| TNF-α | 13.4 | 8.1 | 3.5 | 42.7 | 11.2 | 54 | ≤ 7.2 |

^†^ Obtained from Mayo Clinic Laboratory

**Table 2.** **Day 4 cytokine data (safety population)**

| **Lab parameter (pg/mL)** | **Mean** | **St. Dev.** | **Min.** | **Max.** | **Median** | ***N*** | **Reference value**^†^ |
| --- | --- | --- | --- | --- | --- | --- | --- |
| IL-10 | 37.6 | 39.6 | 5.9 | 222.2 | 25.5 | 56 | ≤ 6.7 |
| IL-1β | 10.0 | 64.3 | 1.0 | 486.9 | 1.0 | 57 | ≤ 2.2 |
| IL-4 | 45.0 | 70.2 | 7.3 | 392.8 | 25.3 | 57 | ≤ 2.0 |
| IL-6 | 37.6 | 82.0 | 0.7 | 546.7 | 11.4 | 55 | ≤ 3.0 |
| IL-8 | 30.8 | 38.8 | 2.2 | 214.1 | 18.1 | 55 | ≤ 2.8 |
| TNF-α | 12.7 | 11.4 | 0.6 | 52.6 | 8.5 | 54 | ≤ 7.2 |

^†^ Obtained from Mayo Clinic Laboratory

The mean absolute change ranged from -13.9 pg/mL (reported for IL-4) to an increase of 1.3 pg/mL (reported for IL-6), while the mean percent change ranged from -51.4% (reported for IL-1β) to +3.6% (reported for IL-6).

**Table 3.** **Changes in cytokine levels from pre-treatment day 1 to day 4 (safety population)**

| **Lab parameter (pg/mL)** | **Mean absolute change** | **% change of population mean** | ***N*** |
| --- | --- | --- | --- |
| IL-10 | -4.9 pg/mL | -11.5% | 56 |
| IL-1β | -10.6 pg/mL | -51.4% | 57 |
| IL-4 | -13.9 pg/mL | -23.6% | 57 |
| IL-6 | 1.3 pg/mL | + 3.6% | 55 |
| IL-8 | -1.7 pg/mL | - 5.2 % | 55 |
| TNF-α | -0.7 pg/mL | - 5.5% | 54 |

***Other laboratory parameters.*** Mean levels of bilirubin, creatinine, CRP, LDH, BUN, and fibrinogen decreased from baseline (prior to the first treatment) to after the last treatment (Table 4).

**Table 4. Analyte levels from patients who received ≥ 4 treatment cycles (N = 90), subdivided by vital status at day 28**

| **Analyte  (number of patients with data)*** | **Average baseline levels** | | **Average levels after 4th treatment  Value (% change)^†^  reduction (-) / increase (+)** | | **Reference range**** |  |
| --- | --- | --- | --- | --- | --- | --- |
|  | **Alive** | **Deceased** | **Alive** | **Deceased** |  |  |
| BUN (mg/dL) (n=81) | 93.1 | 130.1 | 80.7 (-2.5%) | 114.8 (5.7%) | 6-24 mg/dL |  |
| Creatinine (mg/dL) (n=81) | 1.592 | 1.681 | 1.132 (-17.9%) | 1.120 (-19.5%) | 0.7-1.3 mg/dL |  |
| LDH (U/L) (n=80) | 521.5 | 717.6 | 387.7 (-20.3%) | 522.7 (-19.4%) | 140-280 U/L |  |
| CRP (mg/L) (n=81) | 129.96 | 143.13 | 60.81 (-40.7%) | 102.27 (0.2%) | < 10 mg/dL |  |
| Fibrinogen (mg/dL) (n=82) | 614.5 | 618.6 | 412.0 (-24.9%) | 415.5 (-30.1%) | 200-400 mg/dL |  |
| WBC (10^3^/µL) (n=83) | 14.43 | 12.79 | 16.81 (29.4%) | 14.90 (31.3%) | 4.5 -11 x 10^3^/µL |  |
| Neutrophils (10^3^/µL) (n=54) | 13.07 | 10.22 | 13.40 (24.5%) | 12.35 (22.4%) | 2.5-7 x 10^3^/µL |  |
| Lymphocytes (10^3^/µL) (n=53) | 0.79 | 0.73 | 1.01 (53.0%) | 0.66 (32.9%) | 1-4.8 x 10^3^/µL |  |
| Neutrophil-to-Lymphocyte ratio (NLR)^‡^ (n=50) | 22.85 | 30.07 | 16.95 (25.9%) | 27.05 (85.1%) | 08-3.5 |  |
| * Only patients with values at both baseline and after the 4th treatment were included.  † Calculation of percent change excludes subjects with baseline values of zero.  ‡ NLR excludes subjects with lymphocyte values of zero.  ** From Mayo Clinic Laboratories: www.mayocliniclabs.com | | | | | | |

**Appendix E – Safety data**

**Serious Adverse Events by relationship to study procedure in the treated population (*N*=107)**

|  | **38 Reports** | | | |
| --- | --- | --- | --- | --- |
| **Adverse Event** | **Definitely not related** | **Possibly related** | **Definitely related** | **Unknown** |
| Acidosis | 1 | 0 | 0 | 0 |
| Arrhythmia | 1 | 0 | 0 | 0 |
| Cardiac arrest | 5 | 0 | 0 | 0 |
| Cardiogenic shock | 1 | 0 | 0 | 0 |
| Cardiopulmonary arrest | 3 | 0 | 0 | 0 |
| Clotted AV fistula | 1 | 0 | 0 | 0 |
| Deep vein thrombosis | 1 | 0 | 0 | 0 |
| Hemorrhage requiring mediastinal wash-out | 1 | 0 | 0 | 0 |
| Myocardial infarction | 1 | 0 | 0 | 0 |
| Oxygen desaturation | 3 | 1 | 0 | 0 |
| Pneumomediastinum | 1 | 0 | 0 | 0 |
| Pneumothorax | 2 | 0 | 0 | 0 |
| Respiratory failure | 3 | 0 | 0 | 0 |
| Right ventricular thrombus | 1 | 0 | 0 | 0 |
| Seizure | 0 | 1 | 0 | 0 |
| Suspected bowel perforation | 1 | 0 | 0 | 0 |
| Volume overload | 0 | 0 | 9 | 0 |
| Worsening anemia requiring transfusion | 1 | 0 | 0 | 0 |
| **Total** | **27** | **2** | **9** | **0** |

**Appendix F - Propensity score-matched analysis**

**Demographics and baseline characteristics of propensity-matched patients**

| **Pair/Triplet Number** | **Treatment** | **Age** | **Gender** | **Race / Ethnicity** | **BMI** | **BMI Category** | **History of Cardiac Disease (CAD, CHF, NICM)** | **History of Pulmonary Disease (COPD, Asthma, ILD, pHTN)** | **History of Diabetes** | **History of Hypertension** | **History of Solid Organ Transplant (liver, kidney, lung, heart)** | **Ventilator** | **Date of Hospital Admission** | **Date of COVID ICU Admission** |
| --- | --- | --- | --- | --- | --- | --- | --- | --- | --- | --- | --- | --- | --- | --- |
| 1 | MTx | 52 | M | Black | 32.8 | Obese | No | No | Yes | Yes | No | Yes | 2020-11-01 | 2020-11-01 |
| 1 | SOC | 36 | F | White/ Asian | 42.1 | MO | No | Yes | No | No | No | Yes | 2021-07-31 | 2021-08-04 |
| 1 | SOC | 73 | M | White/ Asian | 26.4 | OW | No | No | No | Yes | No | Yes | 2021-08-08 | 2021-08-14 |
| 2 | MTx | 57 | M | Hispanic | 41.6 | MO | No | No | Yes | Yes | No | Yes | 2020-09-06 | 2020-09-06 |
| 2 | SOC | 52 | M | Black | 28.6 | OW | No | No | Yes | Yes | Yes | Yes | 2020-06-30 | 2020-07-10 |
| 2 | SOC | 34 | M | White/ Asian | 13.18 | UW | No | Yes | No | No | No | Yes | 2021-08-12 | 2021-08-13 |
| 3 | MTx | 61 | F | Hispanic | 48.5 | MO | No | No | No | Yes | No | Yes | 2020-11-18 | 2020-11-22 |
| 3 | SOC | 51 | M | White/ Asian | 29.3 | OW | No | No | No | Yes | No | Yes | 2021-02-25 | 2021-02-25 |
| 4 | MTx | 54 | F | Hispanic | 39.1 | Obese | No | No | Yes | No | No | Yes | 2021-08-01 | 2021-08-12 |
| 4 | SOC | 71 | M | Black | 12.32 | UW | No | Yes | Yes | Yes | Yes | Yes | 2021-01-12 | 2021-01-19 |
| 4 | SOC | 56 | M | White/ Asian | 29.91 | OW | No | No | No | Yes | No | Yes | 2020-12-14 | 2020-12-14 |
| 5 | MTx | 67 | F | Black | 48 | MO | No | No | Yes | Yes | No | Yes | 2020-07-17 | 2020-07-29 |
| 5 | SOC | 68 | M | White/ Asian | 35 | Obese | No | No | No | No | Yes | Yes | 2020-07-14 | 2020-07-18 |
| 5 | SOC | 69 | M | White/ Asian | 26.63 | OW | No | No | No | Yes | No | Yes | 2020-09-07 | 2020-09-07 |
| 6 | MTx | 66 | M | Black | 35.6 | Obese | No | Yes | No | Yes | No | Yes | 2020-11-07 | 2020-11-22 |
| 6 | SOC | 63 | M | White/ Asian | 30.9 | Obese | No | No | No | No | No | Yes | 2021-01-26 | 2021-01-28 |
| 7 | MTx | 47 | F | Black | 29.1 | OW | Yes | No | Yes | Yes | No | Yes | 2020-12-25 | 2020-12-27 |
| 7 | SOC | 52 | F | White/ Asian | 22.08 | Healthy | No | Yes | No | No | No | Yes | 2021-01-10 | 2021-01-10 |
| 8 | MTx | 82 | F | Black | 26.9 | OW | Yes | No | Yes | Yes | No | Yes | 2020-07-29 | 2020-08-03 |
| 8 | SOC | 69 | M | Hispanic | 25.3 | OW | No | No | Yes | No | No | Yes | 2020-06-07 | 2020-06-07 |
| 8 | SOC | 82 | F | Black | 37.6 | Obese | No | No | Yes | Yes | No | Yes | 2020-07-03 | 2020-07-03 |
| 9 | MTx | 37 | F | Hispanic | 42.5 | MO | No | No | No | No | No | Yes | 2020-08-30 | 2020-08-30 |
| 9 | SOC | 56 | F | Hispanic | 21.9 | Healthy | Yes | No | No | No | No | Yes | 2020-11-18 | 2020-11-18 |
| 9 | SOC | 73 | M | White/ Asian | 32.2 | Obese | Yes | No | Yes | Yes | No | Yes | 2021-01-20 | 2021-01-20 |
| 10 | MTx | 39 | M | Hispanic | 60.74 | MO | No | No | No | Yes | No | No | 2021-03-22 | 2021-03-22 |
| 10 | SOC | 59 | F | Hispanic | 28.22 | OW | No | No | No | Yes | Yes | No | 2020-12-12 | 2020-12-12 |
| 10 | SOC | 53 | M | White/ Asian | 35.3 | Obese | No | Yes | Yes | Yes | No | Yes |  | 2020-08-19 |
| 11 | MTx | 71 | M | White/ Asian | 31.7 | Obese | No | No | No | Yes | No | Yes | 2020-12-06 | 2020-12-07 |
| 11 | SOC | 46 | M | Missing/ Unknown/ Other | 41.9 | MO | No | No | Yes | Yes | No | Yes | 2021-01-17 | 2021-01-17 |
| 12 | MTx | 68 | M | Hispanic | 30.4 | Obese | No | No | No | Yes | No | Yes | 2021-01-30 | 2021-02-02 |
| 12 | SOC | 62 | F | Hispanic | 46.1 | MO | No | No | No | Yes | No | Yes | 2021-04-05 | 2021-04-05 |
| 13 | MTx | 73 | M | Hispanic | 29.95 | OW | No | Yes | No | No | No | Yes | 2020-07-15 | 2020-07-21 |
| 13 | SOC | 56 | M | White/ Asian | 30.2 | Obese | No | No | No | No | No | Yes | 2021-08-01 | 2021-08-07 |
| 13 | SOC | 51 | M | White/ Asian | 31.1 | Obese | No | No | Yes | Yes | Yes | Yes | 2021-03-12 | 2021-03-12 |
| 14 | MTx | 68 | F | White/ Asian | 27.7 | OW | Yes | No | Yes | No | No | Yes | 2020-12-30 | 2021-02-05 |
| 14 | SOC | 70 | M | White/ Asian | 30.7 | Obese | Yes | Yes | No | No | No | Yes | 2021-01-11 | 2021-01-12 |
| 14 | SOC | 64 | F | Hispanic | 27.63 | OW | No | No | Yes | No | No | Yes | 2020-07-27 | 2020-07-27 |
| 15 | MTx | 77 | M | Hispanic | 24.28 | Healthy | No | No | Yes | No | No | Yes | 2021-01-06 | 2021-01-08 |
| 15 | SOC | 30 | F | Black | 43.6 | MO | No | No | No | No | No | Yes | 2021-05-18 | 2021-05-21 |
| 16 | MTx | 44 | F | Hispanic | 40 | MO | No | No | No | No | No | No | 2021-06-23 | 2021-06-25 |
| 16 | SOC | 61 | F | Black | 30.9 | Obese | No | No | No | No | No | No | 2021-01-14 | 2021-01-15 |
| 16 | SOC | 70 | M | Hispanic | 19.1 | Healthy | Yes | No | Yes | Yes | No | Yes | 2020-07-27 | 2020-07-27 |
| 17 | MTx | 82 | M | White/ Asian | 24.5 | Healthy | Yes | No | No | No | No | Yes | 2020-11-04 | 2020-11-13 |
| 17 | SOC | 87 | M | Hispanic | 29.7 | OW | Yes | No | No | No | No | No | 2020-10-07 | 2020-10-07 |
| 17 | SOC | 84 | M | White/ Asian | 25.5 | OW | No | No | Yes | Yes | No | No | 2021-08-08 | 2021-08-13 |
| 18 | MTx | 51 | M | Hispanic | 28.5 | OW | No | No | No | No | No | Yes | 2021-01-31 | 2021-01-31 |
| 18 | SOC | 61 | M | Hispanic | 29.1 | OW | Yes | Yes | Yes | Yes | No | Yes | 2021-05-14 | 2021-05-17 |
| 19 | MTx | 77 | F | White/ Asian | 35 | Obese | No | Yes | No | Yes | No | Yes | 2021-08-09 | 2021-08-15 |
| 19 | SOC | 60 | M | Black | 30.2 | Obese | Yes | No | Yes | Yes | Yes | Yes | 2021-07-31 | 2021-08-12 |
| 19 | SOC | 72 | M | White/ Asian | 32.7 | Obese | No | Yes | No | No | No | Yes |  | 2020-12-25 |
| 20 | MTx | 60 | M | White/ Asian | 34.6 | Obese | No | No | No | Yes | No | Yes | 2020-10-27 | 2020-10-27 |
| 20 | SOC | 62 | F | White/ Asian | 38.8 | Obese | Yes | Yes | No | No | No | Yes | 2020-12-08 | 2020-12-13 |
| 21 | MTx | 75 | M | White/ Asian | 26.45 | OW | Yes | No | No | Yes | No | Yes | 2020-10-03 | 2020-10-05 |
| 21 | SOC | 66 | F | Black | 27.41 | OW | No | No | Yes | Yes | No | No | 2020-12-07 | 2020-12-08 |
| 21 | SOC | 73 | M | White/ Asian | 25.6 | OW | Yes | No | No | Yes | Yes | Yes | 2020-12-24 | 2020-12-24 |
| 22 | MTx | 63 | M | Black | 32.9 | Obese | No | No | Yes | Yes | No | Yes | 2021-02-17 | 2021-02-21 |
| 22 | SOC | 32 | M | Hispanic | 30.8 | Obese | No | No | No | No | No | Yes | 2021-02-08 | 2021-02-08 |
| 23 | MTx | 18 | M | Hispanic | 54.9 | MO | No | No | Yes | Yes | No | Yes | 2021-01-20 | 2021-01-20 |
| 23 | SOC | 66 | F | Hispanic | 23.1 | Healthy | No | No | No | Yes | No | Yes | 2021-01-11 | 2021-01-13 |
| 24 | MTx | 35 | M | Hispanic | 43.8 | MO | No | No | No | No | No | Yes | 2021-01-15 | 2021-01-15 |
| 24 | SOC | 70 | F | Hispanic | 58.81 | MO | No | No | Yes | Yes | No | Yes | 2020-10-19 | 2020-10-20 |
| 25 | MTx | 52 | F | Hispanic | 36.1 | Obese | No | No | Yes | No | No | Yes | 2020-05-16 | 2020-05-16 |
| 25 | SOC | 41 | F | Hispanic | 50.3 | MO | No | No | Yes | Yes | No | Yes | 2020-06-07 | 2020-06-14 |
| 25 | SOC | 61 | M | Hispanic | 22.8 | Healthy | No | No | No | No | No | Yes | 2020-01-27 | 2020-01-27 |
| 26 | MTx | 45 | M | Hispanic | 23.6 | Healthy | No | No | No | No | No | Yes | 2021-04-30 | 2021-05-05 |
| 26 | SOC | 57 | M | Hispanic | 39.6 | Obese | Yes | No | Yes | Yes | Yes | Yes | 2021-06-11 | 2021-06-14 |
| 27 | MTx | 71 | F | White/ Asian | 18.8 | Healthy | No | No | Yes | Yes | No | Yes | 2021-02-02 | 2021-02-02 |
| 27 | SOC | 58 | F | Black | 24.1 | Healthy | Yes | Yes | Yes | Yes | No | Yes | 2021-01-02 | 2021-01-04 |
| 27 | SOC | 42 | M | White/ Asian | 35 | Obese | No | No | No | No | No | Yes | 2021-07-11 | 2021-07-13 |
| 28 | MTx | 82 | M | Black | 26.9 | OW | No | No | No | No | No | Yes | 2021-01-10 | 2021-01-11 |
| 28 | SOC | 83 | M | Hispanic | 34.3 | Obese | No | No | No | Yes | No | Yes | 2021-07-30 | 2021-08-05 |
| 29 | MTx | 71 | M | White/ Asian | 29.4 | OW | Yes | No | No | Yes | Yes | Yes | 2021-01-31 | 2021-01-31 |
| 29 | SOC | 64 | F | Hispanic | 51.2 | MO | Yes | No | Yes | Yes | No | Yes | 2020-11-21 | 2020-11-25 |
| 30 | MTx | 70 | F | White/ Asian | 26.93 | OW | No | No | No | Yes | No | No | 2021-01-18 | 2021-01-22 |
| 30 | SOC | 85 | M | White/ Asian | 20.7 | Healthy | No | No | No | No | No | Yes | 2020-11-17 | 2020-11-21 |
| 30 | SOC | 36 | M | Hispanic | 33 | Obese | No | Yes | Yes | Yes | No | No | 2020-11-14 | 2020-11-16 |
| 31 | MTx | 55 | M | Hispanic | 30.67 | Obese | Yes | Yes | Yes | Yes | Yes | No | 2021-05-30 | 2021-06-11 |
| 31 | SOC | 46 | M | Missing/ Unknown/ Other | 20.9 | Healthy | Yes | No | No | Yes | Yes | Yes | 2021-01-14 | 2021-01-14 |
| 31 | SOC | 81 | F | Hispanic | 36.8 | Obese | No | No | Yes | No | No | No | 2021-01-12 | 2021-01-13 |
| 32 | MTx | 66 | M | Hispanic | 36.5 | Obese | No | No | No | No | No | Yes | 2020-12-30 | 2021-01-03 |
| 32 | SOC | 68 | M | Hispanic | 37.6 | Obese | No | No | Yes | Yes | No | Yes |  | 2021-01-18 |
| 33 | MTx | 75 | M | White/ Asian | 24.17 | Healthy | Yes | No | No | Yes | No | No | 2021-10-29 | 2021-10-30 |
| 33 | SOC | 66 | F | White/ Asian | 36.3 | Obese | Yes | Yes | No | No | No | Yes | 2020-05-14 | 2020-06-06 |
| 33 | SOC | 77 | M | Missing/ Unknown/ Other | 17 | UW | Yes | Yes | No | No | No | No |  | 2020-11-10 |
| 34 | MTx | 76 | M | White/ Asian | 36.4 | Obese | Yes | No | Yes | Yes | Yes | Yes | 2020-12-15 | 2020-12-16 |
| 34 | SOC | 43 | F | Hispanic | 43.5 | MO | Yes | No | Yes | Yes | No | Yes | 2020-07-27 | 2020-07-31 |
| 34 | SOC | 32 | F | Hispanic | 47.5 | MO | Yes | No | Yes | Yes | No | Yes | 2020-07-21 | 2020-07-21 |
| 35 | MTx | 62 | M | Black | 28.35 | OW | No | Yes | No | Yes | Yes | Yes | 2020-10-16 | 2020-10-21 |
| 35 | SOC | 53 | M | Hispanic | 35 | Obese | No | No | No | No | No | Yes | 2021-04-19 | 2021-04-23 |
| 36 | MTx | 58 | M | White/ Asian | 35.59 | Obese | Yes | Yes | Yes | Yes | No | Yes | 2020-09-23 | 2020-09-24 |
| 36 | SOC | 36 | F | Hispanic | 38.1 | Obese | No | No | Yes | No | No | No | 2020-11-27 | 2020-11-29 |
| 36 | SOC | 70 | M | Black | 22.8 | Healthy | No | No | No | Yes | No | No | 2021-03-22 | 2021-03-22 |
| 37 | MTx | 61 | M | White/ Asian | 26.3 | OW | No | No | No | Yes | No | Yes | 2020-10-12 | 2020-10-13 |
| 37 | SOC | 72 | F | Hispanic | 29.8 | OW | Yes | No | Yes | Yes | No | Yes | 2021-01-20 | 2021-01-20 |
| 37 | SOC | 70 | F | White/ Asian | 36.5 | Obese | No | Yes | No | Yes | No | Yes | 2021-02-25 | 2021-03-07 |
| 38 | MTx | 60 | M | Hispanic | 24.5 | Healthy | Yes | No | Yes | Yes | Yes | Yes | 2020-07-30 | 2020-07-30 |
| 38 | SOC | 65 | F | White/ Asian | 33.57 | Obese | No | No | No | Yes | No | No | 2021-01-10 | 2021-01-11 |
| 38 | SOC | 62 | F | White/ Asian | 22.7 | Healthy | No | No | No | No | No | Yes | 2020-07-08 | 2020-07-10 |
| 39 | MTx | 59 | M | White/ Asian | 35.6 | Obese | Yes | Yes | No | Yes | Yes | Yes | 2021-01-15 | 2021-01-17 |
| 39 | SOC | 82 | M | Missing/ Unknown/ Other | 26.8 | OW | No | No | No | No | No | Yes |  | 2020-11-04 |
| 40 | MTx | 61 | F | Black | 41.9 | MO | No | No | Yes | Yes | No | No | 2020-08-09 | 2020-08-09 |
| 40 | SOC | 64 | F | Black | 41.8 | MO | No | No | Yes | Yes | No | No | 2020-07-15 | 2020-07-16 |
| 40 | SOC | 84 | M | White/ Asian | 35.5 | Obese | No | No | No | No | No | No | 2020-12-22 | 2020-12-22 |
| 41 | MTx | 75 | M | Missing/ Unknown/ Other | 23.7 | Healthy | No | Yes | No | Yes | No | Yes | 2021-10-06 | 2021-10-12 |
| 41 | SOC | 42 | M | Black | 38.9 | Obese | No | No | Yes | No | No | Yes | 2020-07-04 | 2020-07-04 |
| 41 | SOC | 81 | M | White/ Asian | 24.2 | Healthy | No | No | No | Yes | No | Yes | 2021-01-14 | 2021-01-17 |
| 42 | MTx | 45 | F | White/ Asian | 45.1 | MO | No | Yes | Yes | Yes | Yes | Yes | 2020-12-28 | 2020-12-28 |
| 42 | SOC | 73 | M | Hispanic | 31.8 | Obese | Yes | No | Yes | Yes | No | Yes | 2020-12-22 | 2020-12-28 |
| 42 | SOC | 57 | F | Black | 34.3 | Obese | No | No | No | No | No | Yes |  | 2020-10-16 |
| 43 | MTx | 63 | M | White/ Asian | 34.7 | Obese | Yes | No | No | Yes | No | Yes | 2020-11-12 | 2020-11-27 |
| 43 | SOC | 67 | M | White/ Asian | 30.3 | Obese | Yes | No | Yes | No | No | Yes | 2020-10-08 | 2020-10-12 |
| 43 | SOC | 67 | F | Black | 53.59 | MO | No | No | Yes | Yes | No | Yes | 2020-09-27 | 2020-09-28 |
| 44 | MTx | 74 | M | White/ Asian | 28.1 | OW | No | No | No | No | No | Yes | 2021-01-22 | 2021-01-22 |
| 44 | SOC | 62 | M | White/ Asian | 19.8 | Healthy | No | Yes | No | No | No | Yes | 2021-02-04 | 2021-02-04 |
| 45 | MTx | 65 | M | White/ Asian | 26.2 | OW | No | Yes | No | No | No | Yes | 2020-05-23 | 2020-05-24 |
| 45 | SOC | 39 | M | Hispanic | 26.5 | OW | No | No | No | No | No | Yes | 2020-03-24 | 2020-03-27 |
| 45 | SOC | 74 | F | White/ Asian | 21.1 | Healthy | No | Yes | No | Yes | No | Yes | 2020-07-31 | 2020-07-31 |
| 46 | MTx | 46 | M | Hispanic | 44.05 | MO | No | Yes | No | No | No | Yes | 2020-06-01 | 2020-06-01 |
| 46 | SOC | 66 | F | Hispanic | 41.7 | MO | No | No | Yes | Yes | No | Yes | 2020-06-02 | 2020-06-02 |
| 46 | SOC | 50 | F | Black | 35.24 | Obese | No | No | No | No | No | No | 2020-11-25 | 2020-11-25 |
| 47 | MTx | 52 | M | Hispanic | 33.1 | Obese | No | No | No | No | Yes | Yes | 2021-01-06 | 2021-01-06 |
| 47 | SOC | 55 | M | Black | 29.5 | OW | No | No | No | No | No | Yes | 2021-07-31 | 2021-07-31 |
| 48 | MTx | 67 | F | Hispanic | 61.4 | MO | No | No | Yes | Yes | No | Yes | 2020-07-21 | 2020-07-26 |
| 48 | SOC | 71 | M | White/ Asian | 34.9 | Obese | Yes | No | Yes | Yes | Yes | Yes | 2021-01-06 | 2021-01-08 |
| 49 | MTx | 69 | F | Hispanic | 24.1 | Healthy | No | No | Yes | Yes | No | No | 2020-12-03 | 2020-12-06 |
| 49 | SOC | 61 | M | White/ Asian | 37.6 | Obese | Yes | No | No | Yes | No | Yes | 2020-07-13 | 2020-07-14 |
| 49 | SOC | 68 | M | Hispanic | 25.9 | OW | No | No | No | Yes | Yes | Yes |  | 2020-06-20 |
| 50 | MTx | 27 | F | Black | 29.7 | OW | Yes | No | No | No | No | Yes | 2021-07-27 | 2021-07-27 |
| 50 | SOC | 69 | M | White/ Asian | 27.2 | OW | Yes | No | No | Yes | No | Yes | 2020-11-25 | 2020-11-28 |
| 51 | MTx | 55 | F | White/ Asian | 22.3 | Healthy | No | No | No | Yes | No | Yes | 2021-10-03 | 2021-10-04 |
| 51 | SOC | 68 | F | White/ Asian | 45.5 | MO | No | No | No | Yes | No | Yes |  | 2020-07-24 |
| 51 | SOC | 78 | M | White/ Asian | 23.1 | Healthy | No | No | No | Yes | No | Yes | 2021-01-14 | 2021-01-22 |
| 52 | MTx | 59 | M | Black | 44.1 | MO | No | No | No | No | No | Yes | 2020-10-18 | 2020-10-18 |
| 52 | SOC | 60 | M | White/ Asian | 29.2 | OW | No | No | No | No | No | Yes |  | 2020-12-08 |
| 53 | MTx | 66 | F | White/ Asian | 23.6 | Healthy | No | No | No | No | No | No | 2021-08-07 | 2021-08-21 |
| 53 | SOC | 72 | F | Black | 30.93 | Obese | Yes | Yes | No | Yes | No | No | 2021-03-03 | 2021-03-04 |
| 53 | SOC | 65 | F | Hispanic | 29.6 | OW | Yes | No | Yes | No | No | Yes | 2020-05-22 | 2020-05-31 |
| 54 | MTx | 63 | M | Missing/ Unknown/ Other | 25.9 | OW | No | No | Yes | Yes | No | Yes |  | 2020-07-20 |
| 54 | SOC | 69 | F | White/ Asian | 46.6 | MO | Yes | No | Yes | Yes | No | Yes | 2020-11-01 | 2020-11-03 |
| 54 | SOC | 66 | M | White/ Asian | 17.6 | UW | Yes | No | Yes | Yes | Yes | Yes | 2020-11-27 | 2020-12-01 |
| 55 | MTx | 36 | M | Hispanic | 32.9 | Obese | No | No | No | No | No | Yes | 2020-08-01 | 2020-08-01 |
| 55 | SOC | 69 | F | Hispanic | 32.86 | Obese | No | Yes | Yes | Yes | No | No | 2020-10-03 | 2020-10-09 |
| 55 | SOC | 41 | F | White/ Asian | 20.6 | Healthy | No | No | No | No | No | Yes | 2021-01-20 | 2021-01-21 |
| 56 | MTx | 54 | F | Black | 33.9 | Obese | No | No | No | Yes | No | Yes | 2020-10-14 | 2020-10-14 |
| 56 | SOC | 45 | M | Hispanic | 58.4 | MO | Yes | No | No | Yes | No | Yes | 2020-12-19 | 2020-12-27 |
| 56 | SOC | 42 | M | White/ Asian | 30.74 | Obese | No | No | Yes | No | No | Yes |  | 2021-08-08 |
| 57 | MTx | 26 | M | White/ Asian | 37.7 | Obese | No | No | No | Yes | No | Yes | 2020-11-13 | 2020-11-19 |
| 57 | SOC | 53 | M | Black | 25.4 | OW | No | No | No | Yes | No | Yes | 2020-09-15 | 2020-09-16 |
| 57 | SOC | 48 | M | White/ Asian | 34.8 | Obese | No | No | No | Yes | No | Yes |  | 2020-11-12 |
| 58 | MTx | 61 | M | White/ Asian | 30.3 | Obese | No | No | No | Yes | No | Yes | 2020-12-06 | 2020-12-07 |
| 58 | SOC | 41 | M | Hispanic | 41.6 | MO | Yes | No | Yes | Yes | No | Yes | 2020-10-16 | 2020-10-18 |
| 58 | SOC | 82 | M | White/ Asian | 30.8 | Obese | No | No | No | No | No | Yes | 2021-08-05 | 2021-08-07 |
| 59 | MTx | 41 | F | Hispanic | 35.4 | Obese | No | No | Yes | No | No | Yes |  | 2020-12-24 |
| 59 | SOC | 82 | F | Hispanic | 38.6 | Obese | No | Yes | No | Yes | No | Yes | 2020-10-16 | 2020-10-18 |
| 60 | MTx | 64 | M | Hispanic | 30.4 | Obese | No | No | Yes | Yes | No | No | 2021-01-13 | 2021-01-17 |
| 60 | SOC | 45 | F | White/ Asian | 46.1 | MO | No | No | No | Yes | No | Yes | 2020-11-04 | 2020-11-04 |
| 60 | SOC | 83 | M | Hispanic | 27.5 | OW | No | No | No | Yes | No | No | 2020-10-06 | 2020-10-11 |
| 61 | MTx | 45 | M | White/ Asian | 29.5 | OW | No | No | No | Yes | No | Yes | 2021-01-30 | 2021-01-30 |
| 61 | SOC | 44 | F | Hispanic | 58.8 | MO | No | No | No | No | No | Yes | 2020-11-23 | 2020-11-23 |
| 62 | MTx | 58 | F | Black | 36.7 | Obese | No | No | No | No | No | Yes | 2021-02-17 | 2021-02-20 |
| 62 | SOC | 52 | M | Hispanic | 32.8 | Obese | Yes | No | No | Yes | No | Yes | 2021-02-24 | 2021-02-24 |
| 62 | SOC | 65 | M | Hispanic | 29.8 | OW | No | No | No | Yes | No | Yes | 2020-07-11 | 2020-07-11 |
| 63 | MTx | 77 | M | Hispanic | 32.7 | Obese | Yes | No | No | No | No | Yes | 2021-02-26 | 2021-03-07 |
| 63 | SOC | 42 | M | White/ Asian | 57.8 | MO | No | No | Yes | Yes | No | Yes | 2021-08-05 | 2021-08-05 |
| 64 | MTx | 61 | F | White/ Asian | 32.5 | Obese | No | No | No | No | No | Yes | 2021-08-05 | 2021-08-06 |
| 64 | SOC | 89 | M | Hispanic | 25.3 | OW | Yes | No | No | Yes | No | Yes | 2020-12-05 | 2020-12-05 |
| 65 | MTx | 29 | M | Hispanic | 52.3 | MO | No | No | No | No | No | Yes | 2021-08-16 | 2021-08-16 |
| 65 | SOC | 45 | M | Hispanic | 47.2 | MO | No | No | No | No | No | Yes | 2021-07-31 | 2021-08-01 |
| 66 | MTx | 40 | M | Hispanic | 32 | Obese | No | No | No | No | No | Yes | 2021-09-29 | 2021-09-29 |
| 66 | SOC | 59 | M | Black | 60.3 | MO | No | No | No | Yes | No | Yes | 2021-08-06 | 2021-08-14 |

Abbreviations: MTx, MTx.100 Column; M, male; F, female; MO, Morbidly Obese; OW, overweight; UW, underweight.
